# Strengthened Spin Hall Effect of Circularly Polarized Light Enabled by a Single-Layered Dielectric Metasurface

**DOI:** 10.3390/ma16010283

**Published:** 2022-12-28

**Authors:** Minkyung Kim, Dasol Lee

**Affiliations:** 1 School of Mechanical Engineering, Gwangju Institute of Science and Technology (GIST), Gwangju 61005, Republic of Korea; 2 Department of Biomedical Engineering, Yonsei University, Wonju 26493, Republic of Korea

**Keywords:** spin Hall effect, photonic spin Hall effect, metasurface, circular polarization

## Abstract

The spin Hall effect of light, referring to the spin-dependent and transverse splitting of light at an optical interface, is an interface-dependent phenomenon. In contrast to this commonly accepted statement, it has been recently reported that the spin Hall effect under circularly polarized light is interface-independent. Despite this interface-independence, however, the reflection of the spin Hall shifted beam is mostly suppressed under near-normal incidence, where the spin Hall shift is large because of the handedness reversal that occurs during the reflection. Here we present a single-layered dielectric metasurface to realize the interface-independent and strengthened spin Hall effect of light. Numerical simulation results confirmed that the anisotropic geometry of the metasurface induced phase-reversed reflection for one linear polarization and phase-preserved reflection for the other, thereby strongly strengthening the reflection of the spin-Hall-shifted beam. Our work will pave a route toward the precise displacement of the beam at the nanoscale without perturbing its polarization state.

## 1. Introduction

**Figure 1:**
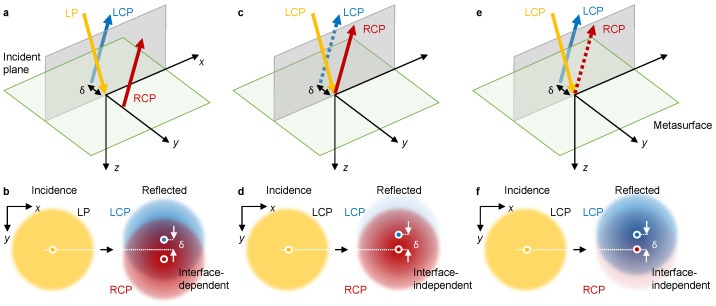
Schematics of the SHEL at an interface between two isotropic media and at the metasurface. LP: linear polarization. (**a**) SHEL and (**b**) beam profiles under linearly polarized incidence at the interface between two isotropic media. (**c**) SHEL and (**d**) beam profiles under circularly polarized incidence at the interface between two isotropic media. (**e**) SHEL and (**f**) beam profiles under circularly polarized incidence at the metasurface, designed to increase the intensity of the spin-Hall-shifted beam by reversing the handedness of the reflected beam.

The spin Hall effect of light (SHEL) is the spin-dependent spatial displacement of light at an optical interface that occurs perpendicularly to the incident plane [1,2,3,4,5]. The transverse and vectorial characteristics of light causes a linearly polarized incidence injected at an interface to split in half into two circularly polarized components along the opposite direction [6,7,8,9,10,11,12] (Figure 1a,b). Because of its microscopic nature, many attempts have been made to amplify the SHEL using various conditions of interfaces, ranging from natural materials [13] to artificially designed media such as multilayers [14,15,16,17], gratings [18], metamaterials [19,20,21,22,23,24,25], and metasurfaces [26,27].

The SHEL under the linearly polarized incidence is intrinsically an interface-dependent phenomenon, in which both the spin Hall shift and efficiency are determined by the Fresnel coefficients of the interface [28]. Therefore, the SHEL has been used to extract unknown information at a given interface, such as the refractive index [29,30], the number of layers [31], the chemical reaction rate [32], the ion concentration [33], etc. [34,35,36], with high precision [37]. In contrast, recent publications have reported that a circularly polarized light injected at an interface that has zero off-diagonal elements of the Jones matrix, i.e., $t_{sp}=t_{ps}=0$ for transmission and $r_{sp}=r_{ps}=0$ for reflection, undergoes an interface-independent SHEL [38,39]. More specifically, the spin Hall shift δ under the circularly polarized incidence ${\vec{e}}_{\pm }=\left({\vec{e}}_{x}\pm i{\vec{e}}_{y}\right)/\sqrt{2}$ is given as [38,39] $\delta^{\pm }=\mp 2\cot\theta_{i}/k_{0}$, where $\theta_{i}$ is the incident angle and $k_{0}$ is the wave vector, for the same handedness and it is given as zero for the opposite handedness. This interface-independent SHEL could be useful for precisely translocating circularly polarized light at the nanoscale by reducing $\theta_{i}$. However, the efficiency of the SHEL under the circularly polarized incidence is still determined by the Fresnel coefficients; unfortunately, the use of a generic interface such as that between two isotropic media reverses the handedness during the reflection process, making the efficiency of the spin-Hall-shifted beam negligible [39] (Figure 1c,d).

The use of an interface between isotropic and anisotropic media satisfying certain conditions has been suggested to increase the efficiency of the shifted beam. The equifrequency curves of the two media cross each other, resulting in the phase reversal of only one polarized component and consequently reversing the handedness of the reflected beam. However, such an interface requires a semi-infinite anisotropic medium, the permittivity of which along the normal vector of the incident plane is less than unity, if the isotropic medium is air. The lack of naturally available materials that satisfy these criteria creates difficulties in the realization of the interface-independent SHEL. Therefore, the proposal of a pragmatic and experimentally feasible interface that supports handedness-preserved reflection under circular polarization, thereby enabling the interface-independent SHEL, is essential (Figure 1e,f).

Here, a single-layered dielectric metasurface consisting of periodically arranged rectangular rods is proposed to strengthen the interface-independent SHEL under circularly polarized incidence. The anisotropic geometry of the designed metasurface breaks the polarization degeneracy, generating a near-zero phase difference between the reflection coefficients for *p* and *s* polarizations under near-normal incidence. The handedness-preserved reflection of circularly polarized incidence at this metasurface enables the reflection of the spin-Hall-shifted beam with an efficiency of more than 65%. This work towards the strengthened, interface-independent SHEL under circularly polarized light provides a path towards the precise displacement of the beam at a nanoscale while keeping its polarization state intact.

## 2. Results and Discussion

### 2.1. Spin Hall Effect under Circularly Polarized Incidence

We start by revisiting the spin Hall shift formula of the reflected beam under an arbitrarily polarized incidence. When the beam waist $w_{0}$ is large enough to satisfy $k_{0}^{2}w_{0}^{2}\gg \cot^{2}\theta_{i}$, an incidence that has the Jones vector of ${\left(\psi_{H},\;\psi_{V}\right)}^{T}$ undergoes the SHEL during the reflection in the following amount [39]:

(1) δ±=∓ReψH∓iψVψHrp∓iψVrsrp+rsk0cotθi,

where the superscript ± represents the handedness of the reflected beam (+ for the left-hand circularly polarized (LCP) and − for the right-hand circularly polarized (RCP) components) and $r_{p}$ and $r_{s}$ are the Fresnel reflection coefficients under *p* and *s* polarizations, respectively. Equation (1) demonstrates that δ of a circularly polarized light ($\psi_{H}=1/\sqrt{2}$ and $\psi_{V}=\pm i/\sqrt{2}$) is $\delta^{\pm }=\mp 2\cot\theta_{i}/k_{0}$, which includes neither $r_{p}$ nor $r_{s}$, for the same handedness and is zero for the opposite handedness. Note that the superscript ± of δ corresponds to the handedness of the incidence, rather than that of the reflected beam. This means that δ is interface-independent and is only determined by the properties of the incidence, i.e., the incident angle and wavelength λ. In particular, as $\theta_{i}$ approaches zero, δ diverges up to [40] $w_{0}/2$, providing a route to the precise control of beam displacement.

In contrast to the interface-independent δ, its efficiency [39],

(2) $$\epsilon^{\pm }={\left|\psi_{H}r_{p}\mp i\psi_{V}r_{s}\right|}^{2}/2,$$

is solely interface-dependent. Note that Equation (2) is valid only under the condition of a large beam waist, as in the spin Hall shift formula presented in Equation (1). By substituting the Jones vector of the circularly polarized light, one can straightforwardly observe that the efficiency of the spin Hall shifted beam is $\epsilon ={\left|r_{p}+r_{s}\right|}^{2}/4$ and that of the handedness-reversed beam is $\epsilon ={\left|r_{p}-r_{s}\right|}^{2}/4$ for both LCP and RCP incidence.

### 2.2. Strategy and Challenges

Meanwhile, under normal incidence, *p* and *s* polarization states are degenerate at an interface between two isotropic media. This polarization degeneracy enforces $r_{p}=-r_{s}$, where the minus sign originates from the sign convention of the Fresnel reflection coefficients [41], making the intensity of the reflected beam that has the same handedness as the incidence zero [39]. This can be also understood as the handedness reversal in the reflection process. Therefore, under near-normal incidence, in which the spin Hall shift is large, the majority of the reflected beam has the opposite handedness with zero spin Hall shift, whereas the efficiency of the spin-Hall-shifted beam is negligibly small. In other words, the interface-independent SHEL under circularly polarized incidence occurs but the isotropy of the media suppresses the reflection of the spin-Hall-shifted beam. Thus, an interface that breaks the polarization degeneracy and thereby supports a large ϵ is required.

**Figure 2:**
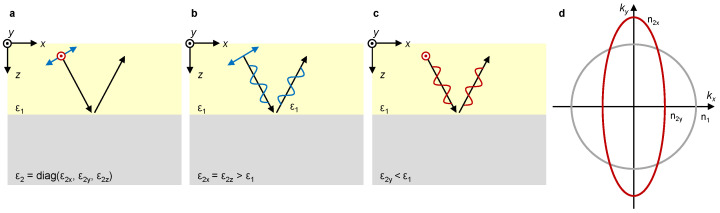
Reflection at an interface between isotropic (permittivity $\varepsilon_{1}$) and anisotropic (permittivity diag($\varepsilon_{2x},\;\varepsilon_{2y},\;\varepsilon_{2z}$)) media. (**a**–**c**) Schematic of the reflection under (**a**) general polarization, (**b**) *p* polarization, and (**c**) *s* polarization. (**d**) Equifrequency curves of the isotropic and anisotropic media, the interface between which supports the handedness-preserved reflection under circularly polarized incidence.

An interface between isotropic and anisotropic uniaxial media that satisfies a certain condition has been suggested as a solution [39] (Figure 2a). If the permittivity of the anisotropic medium along the direction perpendicular to the incident plane is smaller than the permittivity of the isotropic medium while the permittivities along the remaining directions are greater (i.e., $\varepsilon_{2x}=\varepsilon_{2z}>\varepsilon_{1}>\varepsilon_{2y}$ where xz-plane is the incident plane), a circularly polarized incidence reflects while preserving its handedness. Reflection at such an interface under *p*- and *s*-polarized incidence are graphically illustrated in Figure 2b and Figure 2c, respectively. The handedness-preserved reflection of circularly polarized incidence is guaranteed by the phase reversal of *p*-polarized incidence at the sparse-to-dense medium (Figure 2b) and the phase preservation of *s*-polarized incidence at the dense-to-sparse medium (Figure 2c). The equifrequency curves of the two media, the boundaries of which support such handedness-preserved reflection, cross each other, as shown in Figure 2d. However, realizing such anisotropic and semi-infinite medium is challenging.

### 2.3. Metasurface Design and Simulation

**Figure 3:**
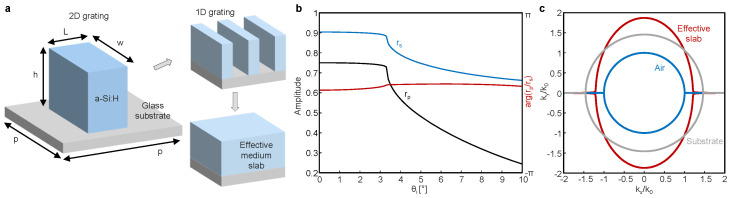
Single-layered dielectric metasurface for the strengthened SHEL under circularly polarized incidence. (**a**) Schematic of the metasurface and its homogenization to 1D grating and slab. (**b**) Reflection coefficients (black for *p* and blue for *s* polarizations, respectively) and their phase difference (red). (**c**) Equifrequency curves of the homogenized metasurface (red), substrate (gray), and air (blue).

Therefore, here we propose a single-layered dielectric metasurface that supports the strengthened and interface-independent SHEL under circularly polarized incidence (Figure 3a). The unit structure of the metasurface is a rectangular rod made with hydrogenated amorphous silicon (a-Si:H) and is periodically arranged on a glass substrate in a square lattice. This single-layered metasurface can be readily fabricated using plasma-enhanced chemical vapor deposition and electron-beam lithography [27]. The reflection coefficients of a given metasurface are calculated by means of rigorous coupled-wave analysis [42]. The geometric parameters of the unit cell are determined via particle swarm optimization to minimize the objective function f=1/(∑Wϵ) under LCP incidence at 633 nm, where $W=\exp(-\theta_{i}^{2}/4)$ is the weight function and the summation runs over from $\theta_{i}=0^{\circ }$ to $10^{\circ }$ with a $1^{\circ }$ step. After 150 iterations, the parameters are given as: periodicity p=418 nm, length L=124 nm, width w=334 nm, and height h=137 nm. The refractive indices of a-Si:H and glass are set as [43] 3.50+0.046i and 1.457, respectively. Compared to the isotropic-anisotropic interface [39], this metasurface is experimentally feasible and compact.

In contrast to the π phase difference between $r_{p}$ and $r_{s}$ at the isotropic–isotropic interface, the anisotropic geometry of the metasurface (L<w) breaks the polarization degeneracy under normal incidence and results in a near-zero phase difference between the reflection coefficients of two linear polarizations (Figure 3b). The phase reversal can also be explained by the effective medium theory. The metasurface, which can be regarded as a two-dimensional (2D) grating, can be approximated to the effective one-dimensional (1D) grating and then to the slab using the zero- and second-order effective medium theory, respectively [44] (Figure 3a, inset). The anisotropic unit structure differentiates the permittivity of the effective slab of *p* and *s* polarizations under normal incidence; the effective index of the homogenized slab is $1.87+6.6\times 10^{-3}i$ for *p* polarization and is $1.21+3.3\times 10^{-3}i$ for *s* polarization. The effective indices of the metasurface are both greater than the index of air but the substrate index is intermediate between them (Figure 3c), reproducing the signature of the equifrequency curves that cross each other (similarly to Figure 2d). Consequently, the near-zero phase difference of $r_{p}$ and $r_{s}$ under normal incidence can be understood as a result of the phase-preserved reflection of the *s*-polarized component at the interface between the effective slab and the substrate.

### 2.4. Spin Hall Effect of Light at the Metasurface

**Figure 4:**
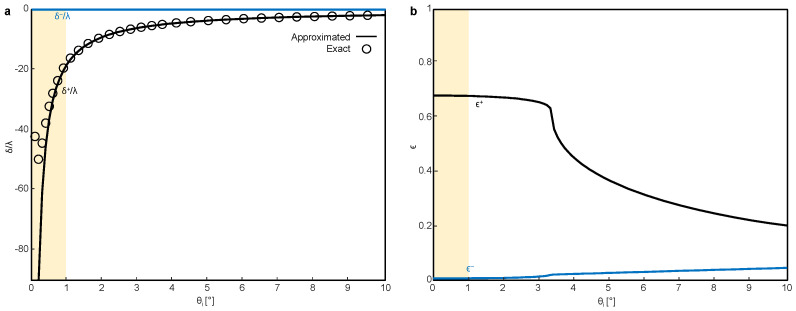
SHEL and its efficiency at the metasurface under LCP incidence. Shaded areas indicate the region in which the large beam waist condition ($k_{0}^{2}w_{0}^{2}\gg \cot^{2}\theta_{i}$) is invalid. (**a**) δ/λ of LCP (black) and RCP (blue) reflected beams. Solid curves and markers indicate results calculated with and without the large beam waist assumption. (**b**) ϵ of the LCP (black) and RCP (blue) reflected beams.

The spin Hall shift and its efficiency at the metasurface under LCP incidence ($\psi_{H}=1/\sqrt{2}$, $\psi_{V}=i/\sqrt{2}$) are numerically examined (Figure 4). For the simulation, we use λ=633 nm, $w_{0}=100\lambda$, and a propagation distance of 10λ. The spin Hall shifts with and without the large beam waist assumption ($k_{0}^{2}w_{0}^{2}\gg \cot^{2}\theta_{i}$) are calculated using Equation (1) and by taking an average of the reflected beam profiles [17], respectively.

As theoretically predicted, an interface-independent spin Hall shift that is nonzero for the same handedness and zero for the opposite handedness ($\delta^{+}=-2\cot\theta_{i}/k_{0}$ and $\delta^{-}=0$) is observed (Figure 4a). At a sufficiently small $\theta_{i}$ ($<1^{\circ }$, denoted by the shaded region in Figure 4a), the large beam waist assumption ($\delta^{\pm }=\mp 2\cot\theta_{i}/k_{0}$) breaks down and $\delta^{+}$ deviates from the cotangent curve and converges to zero (Figure 4a, markers). In this region, the spin Hall shift follows a different, yet still interface-independent formula, $\delta^{\pm }=\mp 2\cot\theta_{i}/k_{0}/(1+4\cot^{2}\theta_{i}/k_{0}^{2}w_{0}^{2}$). In addition, the intensities of the LCP (Figure 4b, black) and RCP (blue) reflected beams clearly prove that the handedness of the reflected beam is reversed. Whereas the efficiency of this spin-Hall-shifted beam at general interfaces is negligible at a small $\theta_{i}$, in which δ is large, the efficiency of the LCP component is significantly improved, reaching 65% at $\theta_{i}<3^{\circ }$. Here, we set the incidence as the LCP, but the same metasurface also operates under the RCP incidence, with $\delta^{+}=0$, $\delta^{-}=2\cot\theta_{i}/k_{0}$ and $\epsilon^{+}$ and $\epsilon^{-}$ being interchanged.

**Figure 5:**
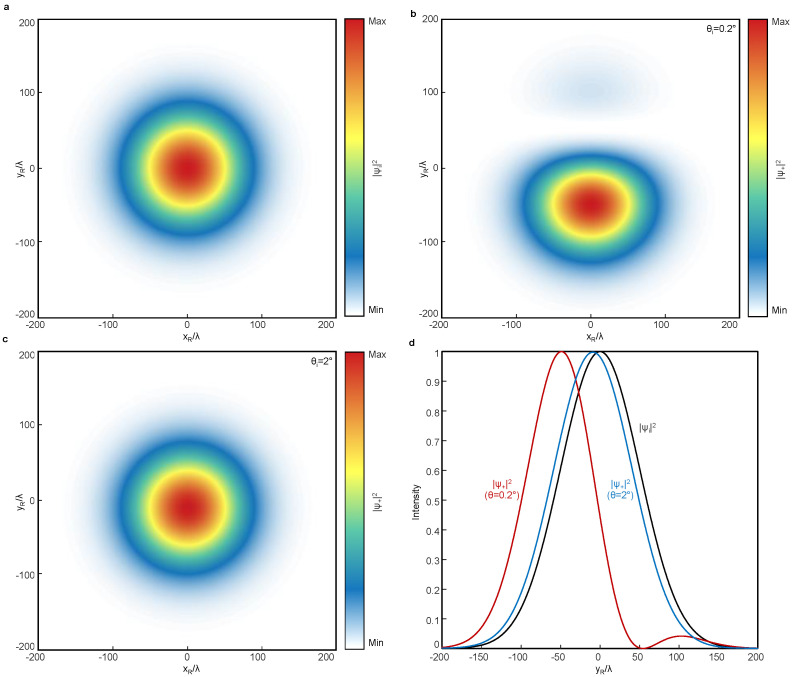
Intensity profiles of the incident and reflected beams. (**a**) 2D intensity profiles of the incident beam. (**b**,**c**) Intensity profiles of the reflected LCP beams at a plane perpendicular to the propagation direction at (**b**) $\theta_{i}=0.2^{\circ }$ and (**c**) $\theta_{i}=2^{\circ }$. (**d**) 1D intensity profile at $x_{R}=0$.

For completeness, the intensity profiles of the incident and reflected beams are presented in Figure 5. All results show the intensity of the LCP components. The Gaussian beam injected at the metasurface (Figure 5a) exhibits a noticeable SHEL along the transverse axis (Figure 5b) while preserving its handedness at $\theta_{i}=0.2^{\circ }$. The 1D intensity profiles along the $y_{R}$-axis at $\theta_{i}=0.2^{\circ }$ indicate that δ reaches approximately $-w_{0}/2$, which is the theoretical limit of the spin Hall shift [40] (Figure 5d). At larger $\theta_{i}$ values, such as that in non-shaded area in Figure 4, the intensity profile of the reflected beam shows a less significant yet nonzero spin Hall shift (Figure 5c,d). These results prove that although the interface-independent SHEL is suppressed in most interfaces because of the handedness reversal nature of reflection, the interface-independent SHEL can indeed be realized using the metasurface.

## 3. Conclusions

In conclusion, a single-layered dielectric metasurface composed of anisotropic rods is proposed to support the strengthened SHEL under circularly polarized incidence. Whereas the interface-independent SHEL under circular polarization is strongly suppressed in generic interfaces under near-normal incidence, the anisotropy of the metasurface results in phase-reversed and phase-preserved reflections for two linear polarizations, respectively, and improve the efficiency of the spin-Hall-shifted beam by more than 65%. Our proposal will find wide applications in controlling circularly polarized light compactly at the nanoscale, while preserving its polarization state.

## Data Availability

Not applicable.

## Abbreviations

The following abbreviations are used in this manuscript:

SHEL	Spin Hall effect of light
LCP	Left circularly polarized
RCP	Right circularly polarized
a-Si:H	Hydrogenated amorphous silicon
